# Items

**Published:** 1866-05

**Authors:** 


THE regular Annual Meeting of the Illinois State Medical Society will be held at Decatur, Tuesday, June 5.

VACCINE VIRUS.—Physicians in the country can obtain healthy and reliable virus by forwarding $1 to R. M. Lackey, M. D., P. O. Box 2175, Chicago.

NEW MEDICAL JOURNALS.—*The Medical and Surgical Monthly*, Memphis, Tenn. Edited by Drs. Ramsey, Saunders, Willett and White.

*The Atlanta Medical and Surgical Journal.* Edited by Dr. J. G. Westmoreland and Dr. W. F. Westmoreland.

Messrs. TICKNOR & FIELDS, of Boston, have placed upon our table their welcome serials for May. The established reputation of the Atlantic Monthly leaves no room for additional praise. *Our Young Folks* is the most readable of all the periodical issues designed for the rising generation; and *Every Saturday* is a weekly which contains, in the form of judicious selections, the cream of current literature.

